# Dual Detection of Hepatitis B and C Viruses Using CRISPR-Cas Systems and Lateral Flow Assay

**DOI:** 10.1155/2024/8819834

**Published:** 2024-10-05

**Authors:** Syeda Najidah Shahni, Sarah Albogami, Iqbal Azmi, Bijay Pattnaik, Rituparna Chaudhuri, Kapil Dev, Jawed Iqbal, Amit Sharma, Tanveer Ahmad

**Affiliations:** ^1^Multidisciplinary Centre for Advanced Research and Studies, Jamia Millia Islamia, New Delhi, India; ^2^Department of Biotechnology, College of Science, Taif University, Taif, Saudi Arabia; ^3^Department Of Pulmanary Medicine and Sleep Disorders, All India Institute of Medical Sciences, New Delhi, India; ^4^Molecular and Cellular Neuroscience, Neurovirology Section, National Brain Research Centre (NBRC), Gurugram 122052, India; ^5^Department of Biotechnology, Jamia Millia Islamia, New Delhi, India

## Abstract

The development of sensitive and specific diagnostic tools for hepatitis B virus (HBV) and hepatitis C virus (HCV) remains crucial for effective disease management and control. In this study, we utilized CRISPR-Cas12 and CRISPR-Cas13 systems for the detection of HBV (DNA virus) and HCV (RNA virus), respectively. We designed and tested multiple guide RNAs (gRNAs) targeting both viruses, confirming successful cleavage of target sequences through gel electrophoresis and a fluorescent reporter assay. Using optimized gRNAs, we developed a lateral flow assay (LFA) for sensitive detection of HBV and HCV, demonstrating a concentration-dependent signal increase. Importantly, no cross-reactivity was observed with other viral targets. To further enhance sensitivity, we employed a dual-enzyme approach, combining Cas12 and Cas13 in a single reaction, which significantly improved detection limits for both viruses. Finally, we developed a dual antigen detection LFA strip capable of simultaneously detecting both HBV and HCV in a single sample. This approach holds promise for point-of-care (POC) diagnostics where the specific viral infection is unknown. This study addresses the current limitations in CRISPR-Cas based diagnostics, namely, the need for ultrasensitive detection methods and the ability to detect multiple antigens using a single test strip. Our findings demonstrate the feasibility of using CRISPR-Cas systems for highly sensitive and specific detection of HBV and HCV, paving the way for potential POC application.

## 1. Introduction

Emerging infectious diseases, like the recent SARS-CoV-2 pandemic, have underscored the critical need for rapid, accurate, and accessible diagnostic tools [1]. While substantial advancements have been made in diagnostic technologies, the development of point-of-care (POC) tests that offer both high sensitivity and cost-effectiveness remains a significant challenge. Clustered regularly interspaced short palindromic repeats (CRISPR)-Cas systems have emerged as a promising tool for nucleic acid detection, with potential applications in POC diagnostics [2, 3]. Their adaptability to various DNA and RNA targets, combined with the possibility of integrating with smartphone technology for analysis, makes them an attractive platform for developing novel diagnostic tests [4, 5]. The CRISPR-Cas system, originally a bacterial adaptive immune system, has been repurposed as a molecular tool for precise detection and cleavage of specific nucleic acid sequences [6, 7]. Different Cas enzymes, such as Cas12 and Cas13, have been harnessed to target DNA and RNA, respectively, enabling the development of versatile diagnostic assays [8, 9]. Recent advancements in CRISPR-Cas diagnostics have led to the development of rapid and accurate tests for a variety of pathogens, including the SARS-CoV-2 virus. These tests have demonstrated high sensitivity and specificity, making them valuable tools in the fight against infectious diseases [10, 11].

Viral hepatitis, caused by hepatotropic viruses like hepatitis A, B, C, D, and E, poses a substantial global health burden. Among these, hepatitis B virus (HBV) and hepatitis C virus (HCV) are particularly concerning due to their propensity to cause chronic infections, leading to severe liver diseases and significant mortality rates [12]. HBV, a DNA virus, and HCV, an RNA virus, infect hundreds of millions worldwide, often progressing unnoticed due to mild or absent symptoms in the early stages [13, 14]. The lack of awareness and delayed diagnosis contribute to the spread of these viruses and the progression to advanced liver disease. Rapid and accurate detection is crucial. While PCR is the gold standard, its requirement for laboratory equipment and trained personnel restricts its use in POC settings. Thus, developing an affordable, rapid, sensitive, and user-friendly diagnostic system remains a significant challenge. Guo et al. recently developed and evaluated a rapid and sensitive method for the simultaneous detection of hepatitis B and C viruses. This method combines reverse transcription loop-mediated isothermal amplification (RT-LAMP) with a lateral flow strip. The amplified products can be visualized on lateral flow bands or measured using a fluorescence detector. The assay demonstrates sensitivity, detecting both HBV and HCV in a single tube and strip within 25 min, with clinical sensitivities of 10 and 10^3^ genomic copies/reaction for HBV and HCV, respectively [15]. Advancements in CRISPR-Cas diagnostics enable enhanced sensitivity and specificity in detection. Utilizing different Cas enzymes, such as Cas12 and Cas13, which possess collateral cleavage activity, significantly contributes to this field.

In this study, we leverage the power of CRISPR-Cas systems to develop a diagnostic platform targeting HBV and HCV. We utilize the Cas12a enzyme for HBV (DNA target) and Cas13a for HCV (RNA target), capitalizing on their specific nucleic acid cleavage capabilities. By designing guide RNAs (gRNAs) that target unique regions within the viral genomes, we aim to achieve highly sensitive and specific detection. In addition to traditional detection methods, we explored the integration of lateral flow assays (LFAs) with smartphone-based analysis, offering a potential route towards user-friendly and portable POC diagnostic.

## 2. Results

### 2.1. CRISPR-Cas12-Based Detection of HBV and Cas13-Based Detection of HCV

**Figure 1:**
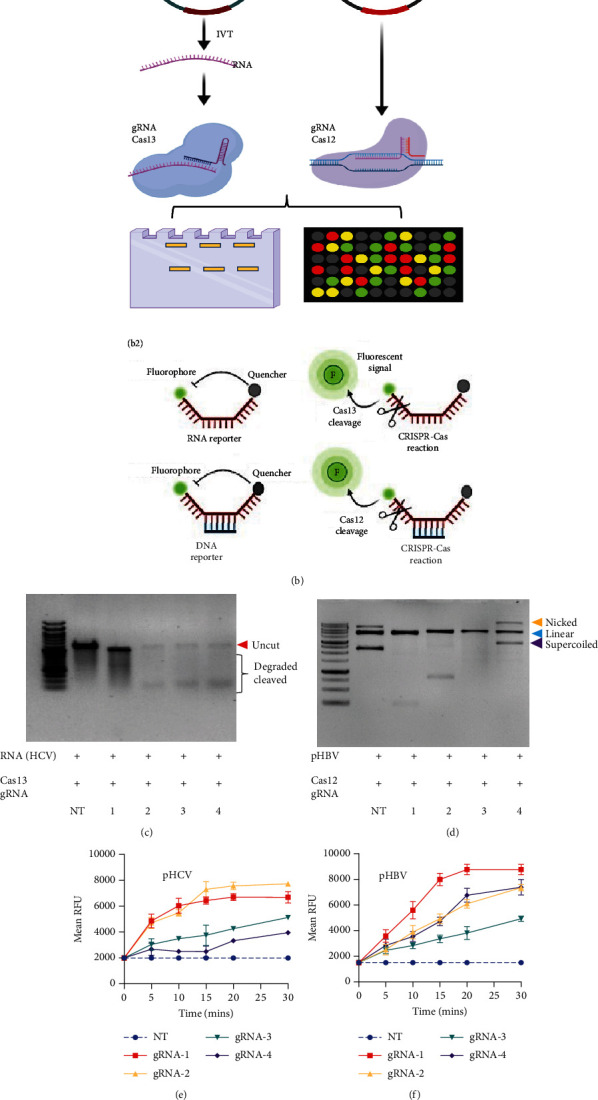
CRISPR-Cas detection of HCV and HBV: (a) A1, A2 Schematic diagram illustrating the target region where the gRNAs bind. (b) B1 Schematic representation of CRISPR-based detection of HBV and HCV; B2 Representation of how reporter molecules work. (c) Gel electrophoresis results of CRISPR-Cas13 cleavage using various guide RNAs; NT denotes the nontarget guide RNA, while 1, 2, 3, and 4 correspond to different guide RNAs targeting HCV. (d) Gel electrophoresis result of CRISPR-Cas13 cleavage with different guide RNAs, NT represents the nontarget guide RNA, 1,2,3 and 4 represents different guide RNAs targeting HBV. (e, f) Fluorescent readout result of cleavage of HCV RNA and HBV DNA to select the best performing guide RNA. (g, h) Dose-response cleavage tests with the best-performing gRNAs showing a concentration-dependent increase in fluorescence over time.

HBV is a DNA virus, whereas HCV contains RNA as its genetic material. To detect these viruses, we utilized CRISPR-Cas12 for HBV and CRISPR-Cas13 for HCV. We designed a series of gRNAs targeting various regions of the HCV and HBV genomes (Figures 1(a) and 1(b) and Table 1). For HCV detection, an in vitro reaction with a plasmid template generated RNA products. These gRNAs were tested against plasmids with the target sequences for HCV and HBV. Gel electrophoresis of the Cas12 and Cas13 protein-gRNA complexes showed clear cleavage of the target plasmids, indicating successful targeting by the gRNAs (Figures 1(c) and 1(d)). We first confirmed this visually and selected the consistent results from the target cleavage. If Cas12 or Cas13 successfully cleaved the DNA or RNA, we should see additional bands, representing the cleaved fragments. To quantify the cleavage, image analysis software can be used to measure the band intensities, ensuring that the selected gRNAs exhibit the highest cleavage efficiency. Based on these results, gRNA-2 for HCV and gRNA-1 for HBV were selected for further experiments. To assess reaction efficiency, we used fluorescent reporter molecules (DNA reporters for Cas12 and RNA reporters for Cas13) and measured fluorescence over time. We observed a gradual increase in fluorescence, confirming the successful detection of HBV DNA with gRNA-Cas12 and HCV RNA with gRNA-Cas13 (Figures 1(e) and 1(f)). Notably, Figure 1(e) shows that gRNA-2 for HCV and gRNA-1 for HBV also exhibited similar cleavage efficiency in the collateral cleavage assay. Based on these consistent results, gRNA-2 for HCV and gRNA-1 for HBV were selected for further experiments. Additionally, dose-response cleavage tests with the best-performing gRNAs showed a concentration-dependent increase in fluorescence over time (Figures 1(g) and 1(h)). Overall, these results demonstrate the CRISPR-Cas system's effectiveness in detecting HBV and HCV, highlighting its potential for sensitive and specific molecular diagnostics.

### 2.2. Optimization of the gRNA for the Detection of HCV and HBV

**Figure 2:**
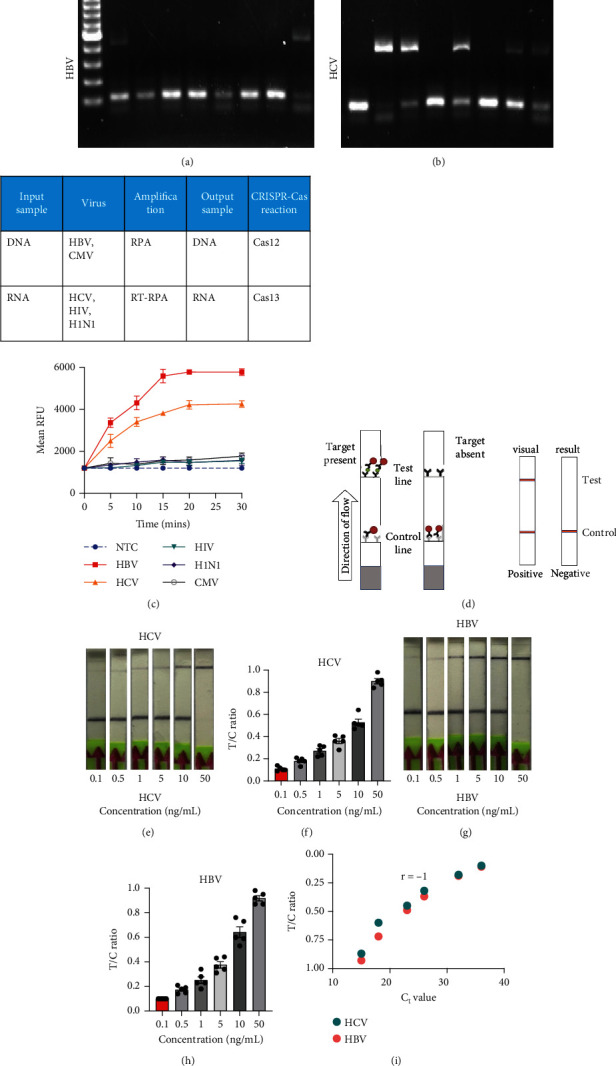
Optimization of the gRNA for the detection of HCV and HBV: (a, b) Gel electrophoresis image of PCR and RPA reaction to evaluate PCR primer efficiency and compare sensitivity with the RPA reaction taking different PCR primers naming PCR-P1,2,3 and 4 and different RPA primers naming as RPA-1,2,3 and 4 for (a) HBV and (b) HCV. (c) Time-dependent fluorescent readout CRISPR-Cas reaction of both HCV and HBV keeping CMV DNA, HIV RNA, and H1N1 RNA as controls to determine assay specificity. (d) Schematic for the lateral flow assay. (e, g) LFA assay for visual detection of HCV and HBV in a dose-dependent manner. (f, h) Quantitative analysis of LFA assay of (d) and (f) using ImageJ. (i) Validation of the LFA signals with qPCR, showing a correlation between the detection of HCV and HBV by qPCR and LFA.

Next, we employed a LFA to assess the sensitivity of HCV and HBV detection. Initially, we screened a set of RPA and PCR primers targeting various regions of the HCV and HBV genomes (Table 2). HCV genome sequences were retrieved from the virus pathogen resource, while HBV sequences were sourced from the HBV database. PCR was conducted to evaluate primer efficiency and comparable sensitivity with the RPA reaction (Figures 2(a) and 2(b)). From this screening, we selected the most effective RPA primers for subsequent reactions. These primers were then used with synthetic DNA for HBV and synthetic RNA for HCV, targeting the screened gRNA. For HCV, RT-RPA followed by IVT was performed to amplify RNA, while RPA was used for HBV, and these samples served as input for the CRISPR-Cas reaction. To determine assay specificity, synthetic fragments containing CMV DNA, HIV RNA, and H1N1 RNA were used as controls. These controls underwent specific RPA reactions and were tested with gRNAs against HBV (for DNA viruses) and HCV (for RNA viruses) using respective reporter systems for Cas12 (DNA reporter) and Cas13 (RNA reporter). A time-dependent increase in fluorescent signals was observed for both HCV and HBV (Figure 2(c)).

Next, we utilized the LFA assay for visual detection of HCV and HBV. To facilitate a clearer understanding of the assay process, a schematic representation of the LFA is provided in Figure 2(d). If the target is absent, the reporter molecule binds to the biotin-streptavidin complex on the control line, producing a visible control line and confirming test validity. If the target is present, the reporter molecule binds to the target analyte, resulting in a visible test line and potentially reducing or eliminating the control line due to decreased binding to the control line. Corresponding synthetic DNA or RNA was used with RPA and RT-RPA for HBV and HCV, respectively, followed by CRISPR-Cas12 and Cas13 reactions. The assay, performed with various concentrations of HBV DNA and HCV RNA, revealed a distinct signal on the LFA test strip with a dose-dependent increase in signal intensity (Figures 2(e), 2(f), 2(g), and 2(h)). Quantitative analysis confirmed the concentration-dependent increase in detection. We validated the LFA signals with qPCR, finding a close correlation between the detection of HCV and HBV by qPCR and LFA (Figure 2(i)). These results demonstrate the effectiveness of the CRISPR-Cas system for detecting HBV and HCV. Importantly, no cross-reactivity was observed with SARS-CoV-2, influenza A, HIV, or CMV, highlighting the assay's specificity.

### 2.3. Dual Enzyme Approach Enhanced the Sensitivity of HBV and HCV Detection

**Figure 3:**
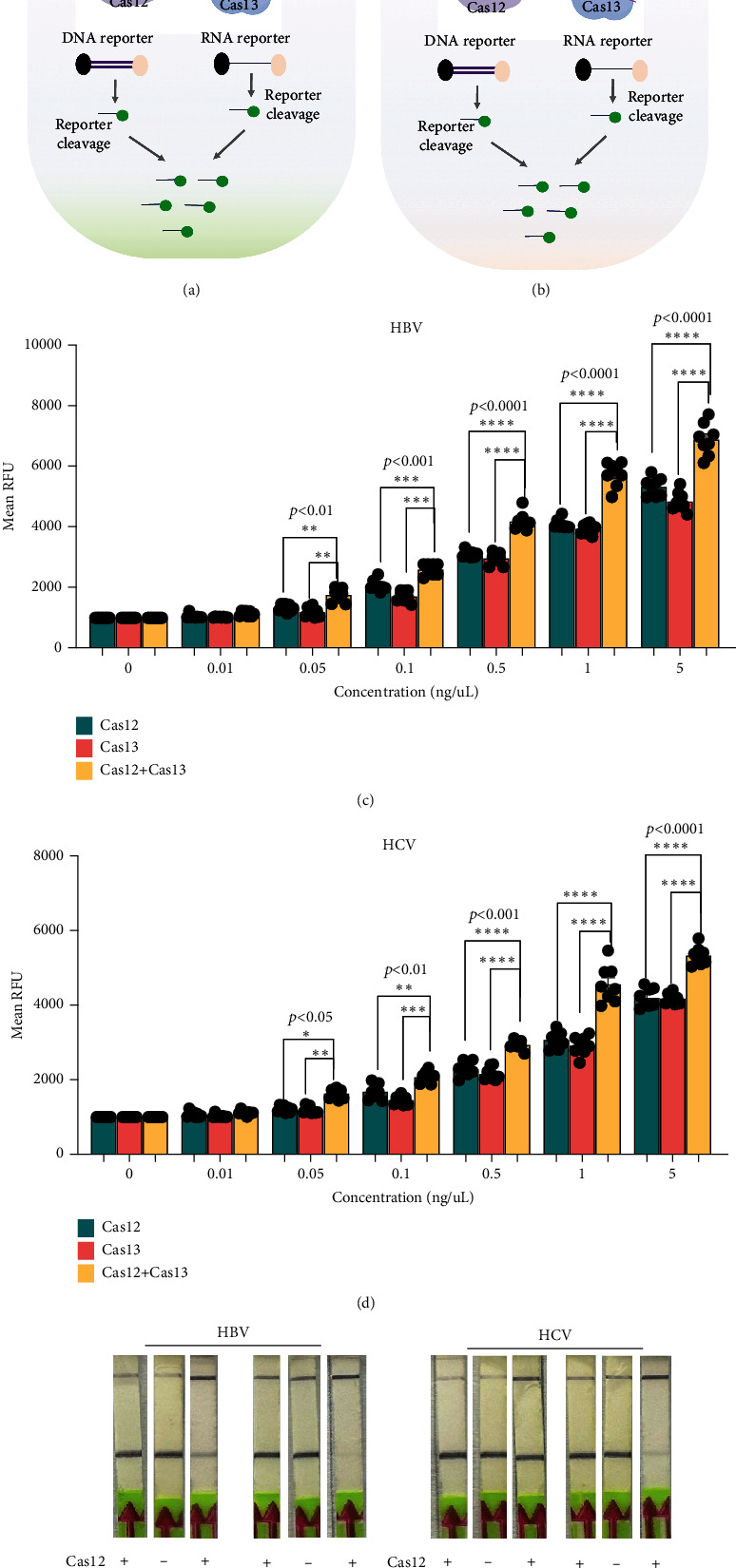
Dual CRISPR-Cas enzyme approach: (a, b) The schematic diagram of the dual-gene detection system for HBV and HCV with Cas12 and Cas13 with FAM DNA and RNA reporters. (c, d) Comparison of fluorescent readout signals of single Cas12 or Cas13 for HBV and HCV with the dual enzyme approach at various concentrations. (e, f) LFA result for comparison of single Cas enzyme reaction of HBV and HCV with the dual Cas enzyme approach at different concentrations.

The main challenge in using CRISPR-Cas tools for molecular diagnostics at the point of care is the lower sensitivity of these assays when combined with LFAs [16]. To enhance sensitivity, we optimized the simultaneous use of Cas12 and Cas13 for detecting HBV or HCV (Figures 3(a) and 3(b)). We used synthetic HCV RNA and HBV DNA at various concentrations in the first reaction and conducted a dual CRISPR-Cas12/13 assay with FAM DNA and RNA reporters. Notably, HCV detection showed significantly higher signal intensity when both Cas12 and Cas13 enzymes were used compared to using a single enzyme (Figure 3(c)). Similarly, the HCV fluorescent signal increased substantially with the dual-enzyme approach compared to using only one enzyme (Figure 3(d)). The signal increase was concentration-dependent, with the dual Cas12 and Cas13 assay detecting as low as 0.05 ng/mL, a sensitivity not achievable by either enzyme alone. Additionally, the LFA signal was significantly stronger with both enzymes compared to individual reactions (Figures 3(e) and 3(f)). These results suggest that the sensitivity of CRISPR-Cas-based detection of HCV and HBV can be enhanced using a dual CRISPR-Cas enzyme approach.

### 2.4. Dual Detection of HCV and HBV in a Single Reaction

**Figure 4:**
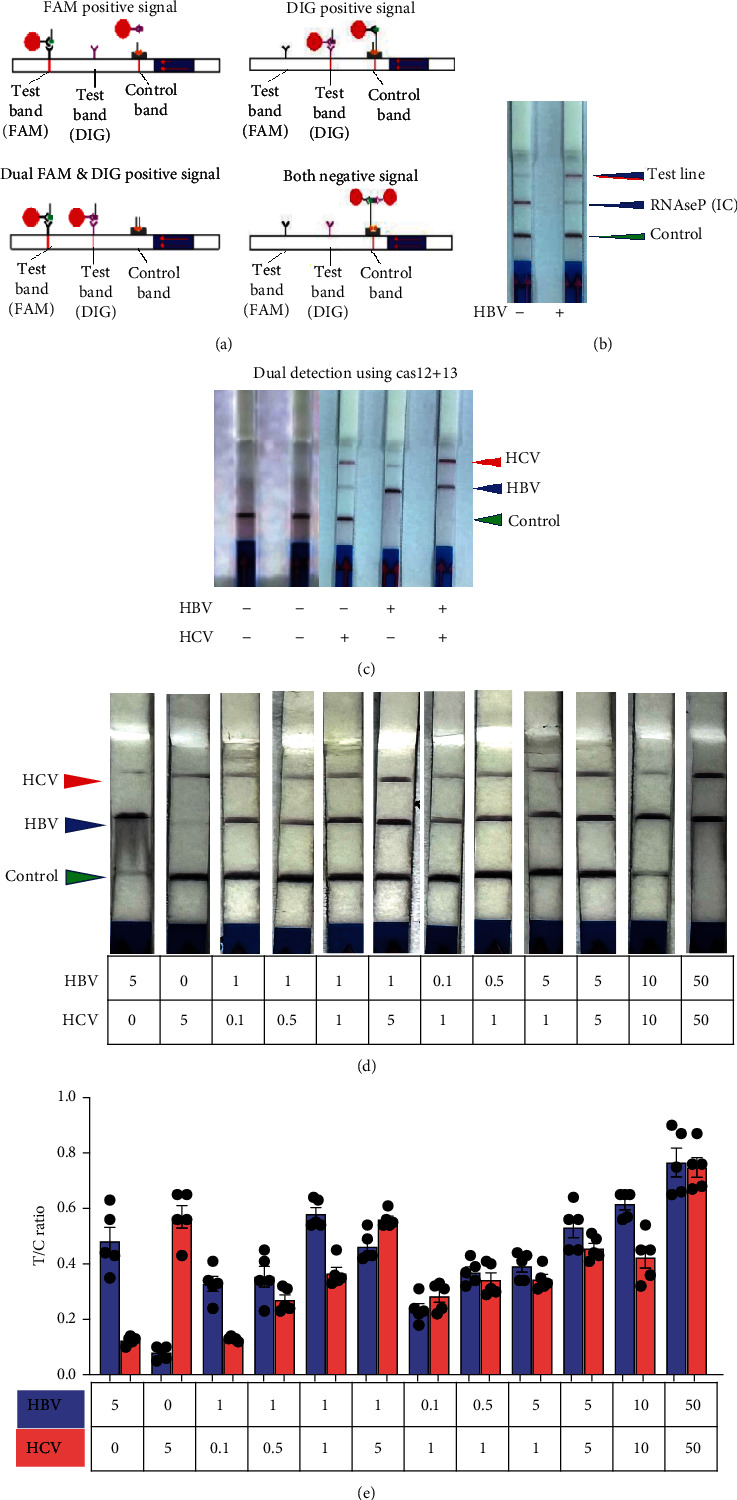
Dual detection of HCV and HBV in a single reaction: (a) Schematic of the fluorescent version of Cas12/Cas13-based assay coupled with lateral flow detection. The intact ssDNA/ssRNA reporter labeled DIG/FAM and biotin flows to the control capture line. When the ssDNA/ssRNA reporter was cleaved by activated Cas12a/Cas13a, the cleaved reporter flows to the target capture line (b). Testing LFA strip for dual detection of HBV in human blood spiked with HBV plasmid and RNAseP as a control in a sample. (c) Dual detection of CRISPR-Cas12/13 for HCV and HBV with the samples containing single HCV or HBV and the sample containing both HCV and HBV. (d) Dual CRISPR-Cas detection of HCV and HBV in different ratios. (e) Quantitative analysis of the robustness of dual detection of HCV and HBV.

To determine if a single LFA strip can detect two antigens simultaneously, we developed a dual antigen detection LFA strip (Figure 4(a)). This strip was designed to detect both FAM and DIG probes concurrently. Initially, we tested the strip for dual detection of HBV and RNaseP in a sample. Human blood spiked with plasmid DNA for HBV produced a clear distinct signal when HBV was absent, detecting only RNaseP in the control sample (Figure 4(b)). Next, we used samples containing both HBV and HCV for detection. As shown in Figures 4(c) and 4(a), distinct signal was obtained for both HBV DNA and HCV RNA when their CRISPR-Cas reactions were performed separately, but the samples were run together on a single LFA strip. Interestingly, distinct signals for both HCV and HBV were observed in the sample containing both reaction mixes, whereas only HCV or HBV was detected in samples with just one of the CRISPR-Cas components for HCV RNA or HBV DNA.

To simplify this as a POC test, we tested various reaction conditions to detect both HCV and HBV in a single sample. We mixed HBV DNA and HCV RNA in different ratios and performed RT-RPA followed by a dual CRISPR-Cas12/13 reaction in the same tube. To mimic physiological conditions, we used normal healthy control blood spiked with HBV DNA and HCV RNA. Surprisingly, both HBV and HCV were detected in a single reaction sample (Figure 4(d)). Quantitative analysis confirmed the robustness of dual detection of HCV and HBV, whether present together in a single sample or alone, indicating the feasibility of this approach for dual antigen detection in situations where the pathogen's exact nature is unknown (Figure 4(e)). The results demonstrated robust detection of either HBV, HCV, or both in samples containing one or both synthetic nucleic acids, supporting the feasibility of dual detection of HCV and HBV in a single sample reaction.

## 3. Discussion

CRISPR-Cas systems have revolutionized molecular diagnostics due to their high specificity, sensitivity, and versatility [17]. Numerous diagnostic assays targeting diverse pathogens, including viruses, bacteria, and parasites, have been developed recently [18, 19]. These assays often utilize the collateral cleavage activity of Cas enzymes (e.g., Cas12 and Cas13) triggered upon target recognition, leading to the amplification of detectable signals [20, 21]. During COVID-19 pandemic, CRISPR-Cas technology has been instrumental in the rapid development of diagnostic tools for SARS-CoV-2 [22]. Leveraging the high specificity and sensitivity of CRISPR-Cas systems, multiple groups across the globe have created assays that can accurately detect SARS-CoV-2 RNA in clinical samples. One of the most notable advancements is the use of Cas13, an RNA-targeting enzyme, which, upon recognizing the viral RNA, activates collateral cleavage of reporter molecules, producing a detectable signal [23]. These assays, such as the SHERLOCK (specific high-sensitivity enzymatic reporter unlocking) and DETECTR (DNA endonuclease-targeted CRISPR trans reporter) platforms, have demonstrated rapid and reliable detection, often within an hour, and can be conducted without the need for sophisticated laboratory equipment [9]. This makes CRISPR-Cas-based diagnostics particularly valuable for POC testing and in resource-limited settings. The versatility of CRISPR technology also allows for the potential multiplex detection of SARS-CoV-2 alongside other respiratory pathogens, enhancing diagnostic capabilities and aiding in better disease management and control during pandemics [22].

In this study, we leveraged both Cas12 and Cas13 to develop an ultrasensitive detection platform for HBV and HCV. Cas12 specifically binds to and cleaves double-stranded DNA, indiscriminately cleaving all DNA molecules present in the system upon activation. We utilized Cas12 to specifically recognize HBV DNA and observed a concentration-dependent increase in the signal in both fluorescent readouts and LFAs. Similarly, Cas13 was employed to specifically recognize HCV RNA. However, individually, neither Cas12 nor Cas13 achieved the sensitivity required to detect these pathogens in clinical samples with very low pathogen loads [24].

To address this, we developed a dual system where both Cas12 and Cas13 were used in a single reaction tube to detect HBV or HCV with much higher sensitivity (0.05 ng/mL) compared to when these enzymes were used individually (0.1 ng/mL). Furthermore, we integrated this dual enzyme reaction with the LFA, which also exhibited higher sensitivity compared to individual enzymes. Thus, this dual enzyme-based reaction provides ultrasensitivity for detecting HCV and HBV. Cas12a has been previously employed for HBV DNA detection, while Cas13a has been utilized for HCV RNA detection, leveraging their distinct collateral cleavage activities to generate detectable signals [25–27]. Various approaches have been explored, including direct detection of viral nucleic acids, isothermal amplification coupled with CRISPR-Cas detection, and integration with LFAs for POC applications [28, 29]. These advancements have demonstrated the feasibility of developing rapid, accurate, and affordable diagnostic tests for HBV and HCV, paving the way for improved disease surveillance and management, particularly in resource-limited settings. However, none of these previous approaches have achieved the sensitivity demonstrated in this study. We successfully achieved this by using both enzymes together in the reaction mix after optimizing various conditions to ensure that the two enzymes did not interfere with each other during the reaction process.

Another challenge with CRISPR-Cas-based molecular diagnostics has been the detection of multiple antigens on a single LFA strip. While some studies have managed to detect multiple antigens using fluorescent and microfluidic systems, this has not been achieved with LFA so far [30]. By leveraging the DNA cleavage activity of Cas12 and the RNA cleavage activity of Cas13, we combined the two systems in a single reaction mix and demonstrated the detection of both pathogens on a single LFA strip. This dual pathogen detection is a significant advancement toward developing CRISPR-Cas-based POC diagnostics and home testing applications. We initially optimized this dual pathogen detection system for HBV and HCV [31]. However, the same approach can be utilized for detecting more similar pathogens where clinical symptoms do not clearly distinguish the nature of the pathogens. One potential future application of our technology is in the detection of respiratory pathogens such as SARS-CoV-2 alongside other pathogens such as SARS-CoV, MERS, or H1N1, which exhibit overlapping clinical symptoms [32]. It must be noted that we obtained a slightly lower sensitivity of the HCV over HBV which could be due to variations in the target HCV RNA sequence or secondary structures that might reduce the CRISPR-Cas efficiency, impacting sensitivity. Further, lower stability and more challenging amplification compared to DNA also plays a role. In our study, HBV was amplified using RPA, directly targeting DNA, while HCV required an additional reverse transcription step (RT-RPA) to convert RNA to DNA. This added complexity likely contributed to the lower sensitivity for HCV detection. Despite SHERLOCK's reported higher sensitivity compared to DETECTR, these RNA-specific challenges might have mitigated its advantages for HCV detection. These factors underscore the need for further optimization to enhance sensitivity across different viral targets, and we have included these considerations in the discussion.

Overall, our study demonstrated the feasibility of using Cas12 and Cas13 in a single reaction to enhance the sensitivity of HCV and HBV detection, which otherwise exhibit low detection sensitivity. Additionally, we showed the utility of Cas12 and Cas13 in detecting HCV and HBV in a single reaction on LFA, broadening the application of CRISPR-Cas POC diagnostics beyond a single pathogen. In the future, we anticipate integrating this dual enzyme and dual pathogen detection system with smartphones for user-friendly applications of CRISPR-Cas diagnostics, as previously demonstrated by us [33].

## 4. Conclusion

In this study, we developed a sensitive and specific method for detecting HCV and HBV simultaneously in a single reaction. This dual CRISPR detection system will enhance the ability to diagnose and manage the patient with coinfection of these viruses. We integrated this dual enzyme reaction with the LFA, which also exhibited higher sensitivity compared to individual enzymes and can be performed faster without the need for a sophisticated laboratory setup making it easy for POC applications.

## 5. Methods

### 5.1. gRNA and Primer Design and Synthesis

To detect HBV and HCV, we designed multiple gRNAs targeting conserved regions of the HBV and HCV genomes (Tables 1 and 2). In this study, we focused on the conserved Core region of HCV genotype 3, which is found across all HCV genotypes. For HBV, we chose a distinct region in the S gene of HBV genotype D to prevent cross-reactivity and false positives that could arise from using highly conserved regions shared among different strains and related viruses. The gRNA sequences were identified using the CHOPCHOP CRISPR-Cas design tool, ensuring specificity and minimal off-target effects. HBV gRNAs were designed for CRISPR-Cas12a (targeting DNA) and HCV gRNAs for CRISPR-Cas13a (targeting RNA). All the sequences were obtained commercially. These primers are synthesized in-house using the online primer 3 design tool or obtained based on previously validated sequences in our lab. The list of the primers is given in Table 3. For HCV, sequences from various genotypes and subtypes were aligned using MUSCLE, focusing on conserved regions to design primers. These primers' properties, such as GC content, melting temperature, and stability, were analyzed using tools like IDT and MFEPrimer 3.0. Similarly, for HBV, sequences from different genotypes were aligned, and primers were designed based on conserved regions. The selected primers were further assessed for their specificity to ensure they could effectively detect the majority of prevalent HCV and HBV genotypes.

Synthetic gene fragments for core (HCV) and S (surface antigen of HBV) genes corresponding to the sequences were designed and purchased commercially. The HCV gene fragments will be synthesized along with the T7 promoter sequence. Similarly, synthetic fragments for CMV, HIV and HIN1 were obtained commercially for the desired target gRNA sequence (Table 2).

### 5.2. RPA and RT-RPA

The RT-RPA methodology involved designing specific primers and probes targeting conserved regions of the HBV and HCV genomes using the methodology described by us previously [33]. For the RNA samples, RT-RPA was performed to amplify the genetic material. The RT-RPA reaction mixture, containing rehydration buffer, magnesium acetate, primers, probe, cDNA template, and nuclease-free water, was incubated at 39°C for 20–40 min. Detection was performed using lateral flow strips or real-time fluorescence monitoring. Specificity was confirmed by testing against nontarget viral nucleic acids, and sensitivity was assessed with serial dilutions of synthetic HBV and HCV RNA. The assay was validated with clinical samples and compared to standard qPCR for accuracy, sensitivity, specificity, and predictive values.

### 5.3. qPCR

For the qPCR methodology, specific primers and probes targeting conserved regions of the HBV and HCV genomes were designed. RNA was reverse transcribed into cDNA using reverse transcriptase, dNTP mix, random hexamers, and reverse transcription buffer. The qPCR reaction mixture included the cDNA template, forward and reverse primers, probe labeled with a fluorophore and quencher, qPCR master mix, and nuclease-free water, totaling 20 *μ*L. The reaction was performed in a qPCR thermocycler (Rotor gene Q, Qiagen) with initial denaturation at 95°C for 5 min, followed by 40 cycles of 95°C for 15 s and 60°C for 1 min as described by us previously [33]. Fluorescence signals were measured at each cycle to quantify the amount of target DNA or RNA. The specificity of the qPCR assay was tested against nontarget viral nucleic acids, and sensitivity was determined using serial dilutions of synthetic HBV and HCV RNA.

### 5.4. Plasmid and RNA Template Preparation

For HBV detection, plasmids containing HBV DNA (genotype D) target sequences were synthesized and cloned into a suitable vector. For HCV (genotype 3) detection, in vitro transcription (IVT) was used to generate RNA templates from plasmid DNA containing HCV target sequences. These templates served as targets for gRNA-Cas complexes during initial validation and optimization experiments.

### 5.5. CRISPR-Cas Reaction Setup

Cas12a and Cas13a proteins were expressed and purified using established protocols. The reactions were set up by mixing Cas12a or Cas13a with their corresponding gRNAs. HBV detection used Cas12a-gRNA complexes, and HCV detection used Cas13a-gRNA complexes. The reactions were incubated with target DNA or RNA sequences in a buffer optimized for CRISPR activity. The reaction was performed as described by us previously [33].

### 5.6. Validation of gRNA Efficacy

The cleavage activity of gRNA-Cas complexes was validated using gel electrophoresis and fluorescent reporter assays. For gel electrophoresis, reaction products were resolved on agarose gels, and successful cleavage was indicated by the presence of expected DNA or RNA fragments. Fluorescent reporter assays employed FAM-labeled reporter molecules (DNA for Cas12 and RNA for Cas13). The fluorescence increase was monitored using a microplate reader, confirming target cleavage and reporter activation.

### 5.7. Optimization of Reaction Conditions

To optimize the sensitivity and specificity of the detection system, we performed titration experiments with varying concentrations of target nucleic acids. The reaction conditions, including incubation time, temperature, and gRNA to target ratio, were optimized to achieve the best performance. The optimal conditions were used in subsequent LFAs.

### 5.8. Dual-Enzyme Reaction and LFA Detection

To enhance detection sensitivity, we combined Cas12a and Cas13a in a single reaction. Synthetic HBV DNA and HCV RNA were mixed with Cas12a and Cas13a complexes, respectively. The dual enzyme reaction was incubated, and the products were applied to the LFA strip (Milenia Biotech). The presence of HBV and HCV was indicated by distinct colored lines on the LFA strip, corresponding to the accumulation of gold nanoparticles at the test and control lines. The specificity of the LFA was assessed using nontarget viral nucleic acids, including CMV DNA, HIV RNA, and H1N1 RNA. These controls ensured no cross-reactivity with nontarget sequences. Sensitivity was determined by testing serial dilutions of synthetic HBV DNA and HCV RNA, with detection limits established by the lowest concentration yielding a visible LFA signal.

### 5.9. Design of the Colorimetric Reporter Molecule

The reporter molecule was developed for colorimetric detection using LFA, based on a previously published method [34]. A short poly-U (hexanucleotides) sequence was synthesized, flanked by FAM and biotin molecules (5′6-FAM/rUrUrUrUrUrU/3 Biotin). Additionally, a DNA reporter for Cas12 was designed. When the activated Cas13a or Cas12a-crRNA complex identified the target sequence, the enzyme cleaved the reporter, releasing FAM/DIG and Biotin/Q moieties. The Biotin/Q was detected in the LFA control lane through its interaction with streptavidin, while the FAM/DIG was detected in two different test lanes.

### 5.10. Quantitative Analysis and Validation

LFA signals were quantified using an ImageJ analysis platform, converting the visual signal into numerical data for comparison with qPCR results as described by us previously [33]. The correlation between LFA and qPCR data validated the accuracy and reliability of the developed assay.

### 5.11. Dual Detection of HBV and HCV in Blood Spiked in Samples

To test the dual detection capability, we used blood serum samples obtained from the healthy donors and spiked with known concentrations of HBV DNA and HCV RNA. Samples were processed and subjected to the dual enzyme reaction followed by LFA detection. The assay's robustness was confirmed by consistent detection of both viruses in mixed and individual samples.

### 5.12. Statistical Analysis

Statistical analysis of the samples was performed using GraphPad Prism 8. The Pearson correlation coefficient was used for correlation analysis. Nonparametric *t* test was used to compare the mean difference between two data sets as mentioned in the results.

## Figures and Tables

**Table 1:** HCV genotype 3 guide RNA for LwaCas13a.

	**Target sequence**	**Guide RNA (5'-3')**
1.	**CTAGCCATGGCGTTAGTACGAGTG**	GAUUUAGACUACCCCAAAAACGAAGGGGACUAAAACCACUCGUACUAACGCCAUGGCUA
2.	**TAGCCATGGCGTTAGTACGAGTGT**	GAUUUAGACUACCCCAAAAACGAAGGGGACUAAAACACACUCGUACUAACGCCAUGGCU
3.	**ACCGGGTCCTTTCTTGGAGCAACC**	GAUUUAGACUACCCCAAAAACGAAGGGGACUAAAACGGUUGCUCCAAGAAAGGACCCGG
4.	**AGCCATGGCGTTAGTACGAGTGTC**	GAUUUAGACUACCCCAAAAACGAAGGGGACUAAAACGACACUCGUACUAACGCCAUGGC

**Table 2:** HBV genotype D guide RNA for LbCas12a and sources of the various viruses used.

	**PAM and Target sequence**	**Guide RNA (5'-3')**
1.	**TTTCCGTCCGAAGGTTTGGTACAGC**	UAAUUUCUACUAAGUGUAGAUGCUGUACCAAACCUUCGGACG
2.	**TTTGTCCTCTGATTCAAGGATCCTC**	UAAUUUCUACUAAGUGUAGAUGAGGAUCCUUGAAUCAGAGGA
3.	**TTTGGTACAGCAACAGGAGGGATAC**	UAAUUUCUACUAAGUGUAGAUGUAUCCCUCCUGUUGCUGUAC
4.	**TTTCTAGGGGGAACTACCGTGTGTC**	UAAUUUCUACUAAGUGUAGAUGACACACGGUAGUUCCCCCUA
5.	HIV	**Reference:** https://www.ncbi.nlm.nih.gov/pmc/articles/PMC7321785/#mmc1
6.	HIN1	https://www.ncbi.nlm.nih.gov/pmc/articles/pmid/35664829/
7.	CMV	https://www.ncbi.nlm.nih.gov/pmc/articles/pmid/37150408/
8.	HCV	https://www.ncbi.nlm.nih.gov/nuccore/EF025301.1
9.	HBV	https://www.ncbi.nlm.nih.gov/nuccore/AB644331.1

**Table 3:** List of RPA and RT-PCR primers for HCV and HBV.

	**Virus**	**Primer Sequences(5'➔3') strand (+) and (-)**	**Size (bp)**	**GC (%)**	**T **_**m**_ ** (°C)**	***Δ*G (kcal/mol)**
RPA primer	**HCV**	**Fwd** (underlined is T7 promoter region) T7-5'-GAAATTAATACGACTCACTATAGGGCCTAGCCATGGCGTTAGTA-3'	41	44	66.5	-56.1
	**HCV**	**Rvs** 5'-TGAGCGGGTTGCTCCAAGAAAGGACCCGGT-3'	30	60	67.1	-46.3

RT-PCR	**HCV**	**Fwd** **5'-**CCTAGCCATGGCGTTAGTAC-3'	20	55	53.8	-25.9
	**HCV**	**Probe** 5'-TGAGTACACCGGAATCGCTGGGGTG-3'	25	60	62.6	-36.7
	**HCV**	**Rvs** 5'-GGTTGCTCCAAGAAAGGACCC-3'	21	57	56.3	-28

RPA primer	**HBV**	**Fwd** 5'-GTTGCCCGTTTGTCCTCTGATTCAAGGATC-3'	30	50	63	-41.1
	**HBV**	**Rvs** 5'-GCAATTTCCGTCCGAAGGTTTGGTACAGCA-3'	30	50	63	-42.9

RT-PCR	**HBV**	**Fwd** 5'- GCCCGTTTGTCCTCTGATTCAAG-3'	23	52	57.1	-30.7
	**HBV**	**Probe** 5'-GCTCAAGGAACCTCTATGTATCCCTCC-3'	27	52	61.3	-35.2
	**HBV**	**Rvs** 5'-TCCGTCCGAAGGTTTGGTACAGC-3'	23	57	58.8	-32.7

## Data Availability

The data utilized in this study are available from the corresponding author upon reasonable request. This includes the raw and processed data used in the analysis.
